# Mechanical and Thermal Properties of Polyurethane Materials and Inflated Insulation Chambers

**DOI:** 10.3390/ma14061541

**Published:** 2021-03-21

**Authors:** Goran Čubrić, Ivana Salopek Čubrić, Dubravko Rogale, Snježana Firšt Rogale

**Affiliations:** 1 Department of Clothing Technology, Faculty of Textile Technology, University of Zagreb, 10 000 Zagreb, Croatia; goran.cubric@ttf.unizg.hr (G.Č.); dubravko.rogale@ttf.unizg.hr (D.R.); sfrogale@ttf.unizg.hr (S.F.R.); 2 Department of Textile Design and Management, Faculty of Textile Technology, University of Zagreb, 10 000 Zagreb, Croatia

**Keywords:** polyurethane, material, clothing, mechanical properties, heat resistance

## Abstract

Evaluating mechanical and thermal characteristics of garment systems or their segments is important in an attempt to provide optimal or at least satisfying levels of comfort and safety, especially in the cold environment. The target groups of users may be athletes engaged in typical sports that are trained in the cold, as well as football players that play matches and train outdoors during the winter season. Previous studies indicated an option to substitute the inner layers of an intelligent garment with polyurethane inflated chambers (PIC) to increase and regulate thermal insulation. In this paper, the authors investigate the mechanical properties of polyurethane material with and without ultrasonic joints. Furthermore, they investigate the potential of designed PICs in terms of efficiency and interdependence of air pressure and heat resistance. The results indicated that an inflated PIC with four diagonal ultrasonic joints has the highest ability to maintain the optimal thermal properties of an intelligent clothing system. The influence of direction and number of ultrasonic joints on the mechanical properties of polyurethane material is confirmed, especially in terms of compression resilience and tensile energy.

## 1. Introduction

The comfort of textile and clothing has been the focus of number investigations, especially the aspects of thermophysiological [1,2,3,4,5] and sensorial comfort [6,7,8,9]. A number of parameters affect the establishment of comfort. According to the comfort equations, parameters are dominantly related to the parameters of body, environment, and clothing. More specifically, the parameters are as follows:

- Parameters of the body: skin wetness (w), skin temperature (t_sk_), DuBois area (A_Du_), the part of skin included in the transfer by radiation (A_r_/A_Du_), and skin emissivity (ε_sk_);
- Parameters of the environment: radiant temperature (t_r_), air velocity (v), air temperature (t_a_), air pressure (p_a_), and pressure on the skin (p_sk,s_);
- Parameters of textile/clothing: clothing insulation (I_cl_), clothing area factor (f_cl_), and clothing temperature (t_cl_).

Furthermore, there are additional parameters of textile materials that affect thermophysiological comfort. Evaluating the thermal properties of garments is crucial for the establishment of optimal or at least satisfying levels of wear comfort, especially when it comes to protective clothing. The evaluation is important for the estimation of the potential heat stress in cold or extremely cold environmental conditions. One of the ongoing missions of researchers is to establish an optimal combination of comfort and thermal insulation to improve garment functionality and sustainability in work and leisure. Clothing has a substantial effect on the thermoregulation of the human body, especially in extreme climatic conditions. Therefore, the thermal comfort of clothing should be precisely defined to ensure optimal use [10]. Clothing with inadequate thermal properties not only diminishes a person’s satisfaction but can also affect their physical performance and may even affect health. Clothing needs to provide optimal insulation in the cold to protect the body from hypothermia. Furthermore, it needs to facilitate the optimal transfer of produced sweat through the garment and the whole clothing system [11].

It is well known that wool is a good regulator in terms of temperature and humidity. Having this in mind, a group of researchers focused on the comparison of outdoor jackets with two different battings in the cold environment, specifically, air temperature of −5 °C and 43% relative humidity [12]. The first batting was made of sheep wool, while the second was made of polyester microfiber. The outcomes of the experiment indicated that the wool batting reduces the chill effect. At the same time, this batting caused an undesirable accumulation of moisture in the jacket, which led to a longer drying rate. Another group of scientists focused on the investigation of the transfer abilities (both heat and mass transport) of single layers through a multilayered system [13]. They measured six characteristics important for the classification of transfer properties. The results showed the heat transfer of the whole system depends on the transfer measured through a single layer. The results of another study indicated a significant influence of fabric finishing on the preservation of heat between inner layers [14]. The performance of multilayer systems changes when the clothing is exposed to various ambient conditions. To investigate this effect, a system consisting of four layers was exposed to seven significantly different ambient conditions, ranging from extremely hot to cold [15]. The authors concluded that exposure to different environmental conditions affects the distribution of moisture accumulated within the layers of the system. In cold, approximately 80% of the moisture was accumulated in the zone of the thermic liner. To assure satisfactory thermal insulation of a garment and increase the protection against cold, inner layers may be replaced by different inflated chambers. The garment can be further improved by the incorporation of activated polyurethane chambers [16]. Further investigation showed that the insulation of a completely activated chamber increases significantly (2.7×) when compared to the chamber before the activation [17]. Over time, the properties of polyurethane material change due to aging. The results of a study focusing on the change in thermal properties of polyurethane-coated material due to natural weathering indicated that, after 3 months of exposure to cold conditions, the thermal resistance decreased by 25% [18].

In this paper, the authors focus on a further investigation of the potential of polyurethane material and polyurethane inflated chambers. The intention is to investigate the mechanical and thermal properties of polyurethane material to design different types of polyurethane inflated chambers, as well as to investigate their efficiency and the interdependence of air pressure and heat resistance.

## 2. Research Methodology

Intelligent thermally adaptive clothes (ITAC) are developed at the Faculty of Textile Technology University of Zagreb [19]. The ITAC activity is based on the integration of air pressure in thermal expanding insulation chambers (TEICs) and sensors for indoor (clothing microclimate) and outdoor (carrier environment) temperature, using a microcontroller with an algorithm and actuators to control the TEIC. When the wearer carries the ITAC, the microcontroller system monitors changes in indoor and outdoor depending on the wearer’s physical activity, compares them with the achieved thermal protection, and makes autonomous decisions on the required degree of thermal protection.

The ITAC has a feature of intelligent clothes because the built-in components automatically follow changes in the clothing microclimate and carrier environment, assess the substantial and necessary state of thermal protection, make decisions, and independently perform the adaptation of thermal insulation to the level ensuring constant thermal comfort. The target groups of users may be sportsmen and recreational athletes engaged in different sports that are trained in the cold, such as skiers, hikers, and sailors, as well as football players that play matches and train outdoors during the winter season. Furthermore, targeted groups include other people doing activities in cold weather conditions [16].

TEIC made of polyurethane foil can replace multiple thermal insulation layers of textile materials. Conventional textile materials have a fixed and constant value of thermal insulation, while TEIC made of highly elastic polyurethane foil can change the value of thermal insulation depending on its filling with air, i.e., thickness. The functional dependencies of changes in the thickness of the TEIC on the air pressure and in the thermal resistance depending on the thickness of the TEIC were investigated. The TEIC was made and integrated into an ITAC and filled with an air pressure of 0–70 mbar, whereby TEIC thicknesses of 0–25 mm were measured. A thermal resistance of 0.188–0.502 m^2^·K·W^−1^ was measured on the thermal manikin. The ratio of thermal insulation of the maximum activated TEIC (thickness 25 mm, air pressure 70 mbar) was almost three times higher than that of the nonactivated TEIC (thickness 0 mm, air pressure 0 mbar).

Therefore, the level of thermal protection depends on the thickness of the TEIC, because there is more air, representing a good insulator, in the thicker chambers. This is the reason why the TEIC was made from airtight highly elastic polyurethane foil. [17].

The TEIC was made of highly elastic polyurethane foil designated as Walopur 4201AU made by Bayer Epurex Films GmbH, Germany. It is characterized by a material density of 1.15 g/cm^3^, a softening point of 140 to 150 °C, a thickness of 0.196 mm, and very high elongation at breaking force, amounting to 550%. Moreover, the material is highly ultraviolet (UV)-resistant and hydrolytically stable, while it has good properties according to thermal and ultrasonic methods, as well as good microbiological stability, which is important for its incorporation into the clothes. The measuring samples of the TEIC were joined using an ultrasonic welding machine.

The research methodology of this paper includes a definition of the selected polyurethane material, measurement of mechanical properties of the polyurethane materials, design of polyurethane insulation chambers (PICs), and a measurement of the heat resistance of inflated polyurethane insulation chambers (PICs). A PIC is an experimental laboratory sample of polyurethane material joined with an ultrasonic machine. Into such a chamber, the air is blown. The blown air provides thermal insulation.

### 2.1. Materials and Joining Technique

**Figure 1:**
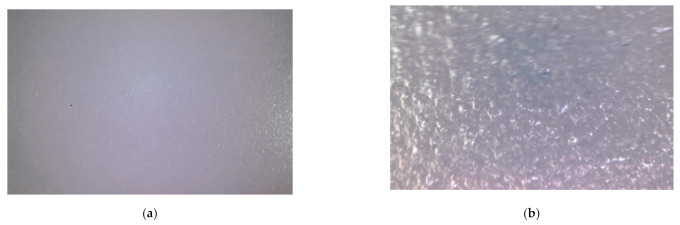
Microscopic image of polyurethane foil: (**a**) under a magnification of 50×; (**b**) under a magnification of 200×.

For the investigation, we selected polyurethane (PU) foil. In Figure 1, microscopic images of the selected foil under a magnification of 50× and 200×, taken by a DINO-Lite microscope, are given. The surface mass of the selected foil was 232 g·m^−2^ with a thickness of 0.25 mm [20].

**Figure 2:**
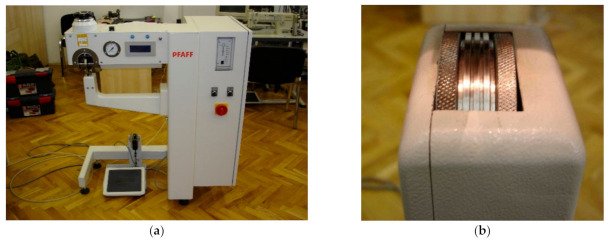
The machine for ultrasonic joining: (**a**) the machine; (**b**) lower engraving counter-roll.

For the preparation of polyurethane material with ultrasonic joints and PICs, an ultrasonic joining machine was used. The machine for ultrasonic joining (Figure 2) had a lower engraving counter-roll, 8 mm wide (the joint was in the form of three lines of the same width). The distance between the lower engraving counter and the sonotrode was set to 0.28 mm. Additionally, the speed of welding was set to 3 m·min^−1^, and the welding energy was 285 W.

### 2.2. Measurement of Mechanical Properties of Polyurethane Materials

For the investigation of mechanical properties, six samples consisting of two layers of polyurethane material were prepared. Five of them were joined ultrasonically. For further reference, prepared samples are designated as follows: PU–number of joints (1, 2, or 4)–the direction of joints (*x*, *y*, or *d*), where the “*y*” direction of joints corresponds to the direction of extrusion of the PU foil, the “*x*” direction corresponds to the direction perpendicular to the extrusion direction, and the “*d*” direction refers to the diagonal direction of joints.

The mechanical properties of polyurethane materials were investigated using a Kawabata Evaluation System (KES). Before testing on the KES system, all samples were conditioned for 24 h (conditions: temperature (T) = 20 ± 2 °C; relative humidity (RH) = 65% ± 2%). All polyurethane materials were investigated in terms of tensile energy (WT), extension (EMT), bending stiffness (B), and shear stiffness (G). The polyurethane material without an ultrasonic joint was additionally tested for compressional energy (WC), compressional resilience (RC), coefficient of friction (MIU), and surface roughness (SMD). A detailed description of measuring techniques was given in previously published papers [20,21].

### 2.3. Design of PICs

All designed PICs consisted of two layers of polyurethane material. PICs were joined ultrasonically. The dimensions of the chambers were 290 × 290 mm, while the dimensions into which the air was blown were 220 × 220 mm. Table 1 shows the PIC dimensions, along with a detailed description.

**Figure 3:**
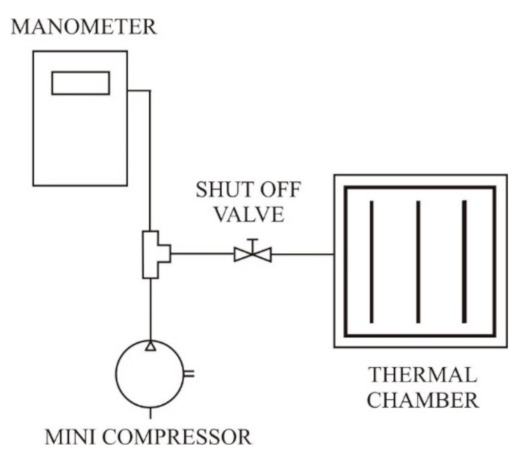
Measuring equipment.

Adequate measuring equipment was designed for inflating air into the PICs (Figure 3). It consisted of a mini compressor, a manometer with a pressure sensor, a shut-off valve, and the PIC. The mini compressor inflated air into the PIC. When appropriate pressure was reached within the PIC, the mini compressor stopped inflating air. The shut-off valve prevented the exit of air from the PIC.

### 2.4. Measurement of Thermal Properties of PICs

**Figure 4:**
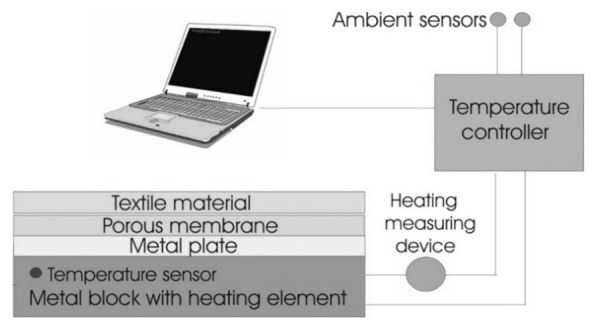
Principle of sweating guarded hotplate (SGHP) device.

For measurement of the heat resistance of designed PICs, a sweating guarded hotplate (model SGHP-8.2) was used. The device simulates the processes of heat and moisture transfer that occur next to the human skin (Figure 4). The measuring device consists of a heated plate with a temperature that matches the skin temperature [22,23].

The heat resistance was calculated using the following equation:

(1) $$R_{ct}=\frac{\left(T_{s}-T_{a}\right)}{\frac{H}{A}}-R_{ct0},$$

where *R_ct_* is the heat resistance of a sample (m^2^·K·W^−1^), *T_s_* is the hotplate surface temperature (°C), *T_a_* is the air temperature (°C), *H*/*A* is the heat flux in the observed zone (W·m^−2^), and *R_ct_*_0_ is the heat resistance of the plate (m^2^·K·W^−1^).

For measurement of the heat resistance, noninflated PICs and PICs inflated at an air pressure of 10, 20, 30, 40, and 50 mbar were prepared.

Testing was conducted at temperatures of 20 °C, 15 °C, and 10 °C. During the testing, the humidity was set to 65% ± 1%. The air velocity was 1 m·s^−1^.

## 3. Results and Discussion

The results include the measured mechanical properties of polyurethane materials and thermal properties of designed PICs.

### 3.1. Measured Mechanical Properties of Polyurethane Materials

**Figure 5:**
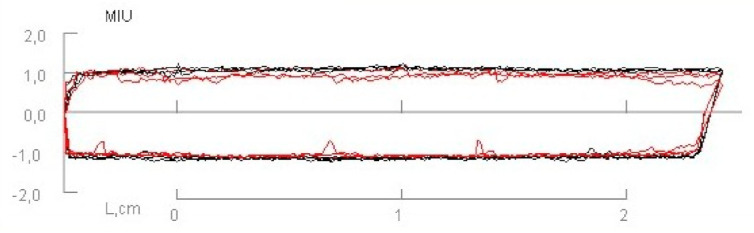
Coefficient of friction of polyurethane material without ultrasonic joint.

**Figure 6:**
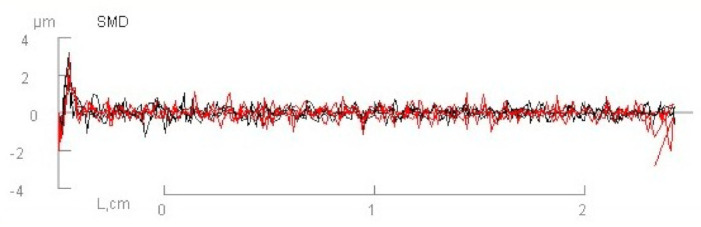
The surface roughness of polyurethane material without ultrasonic joint.

**Figure 7:**
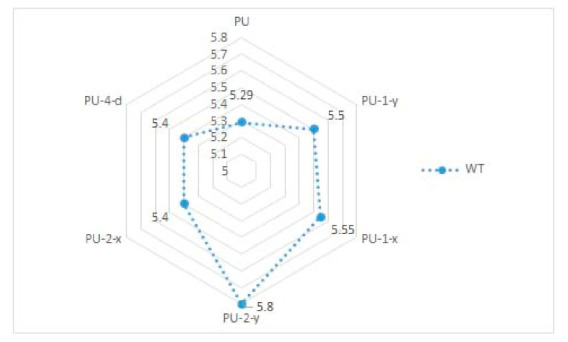
Tensile energy (WT) of polyurethane materials.

**Figure 8:**
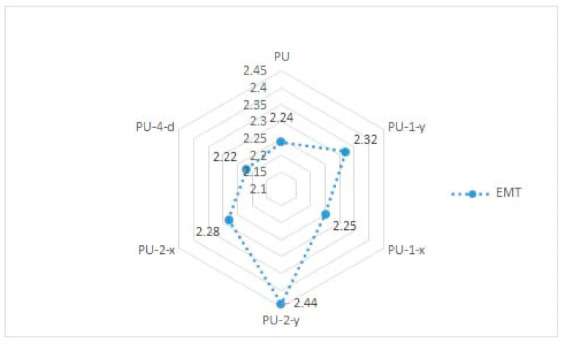
The extension (EMT) of polyurethane materials.

**Figure 9:**
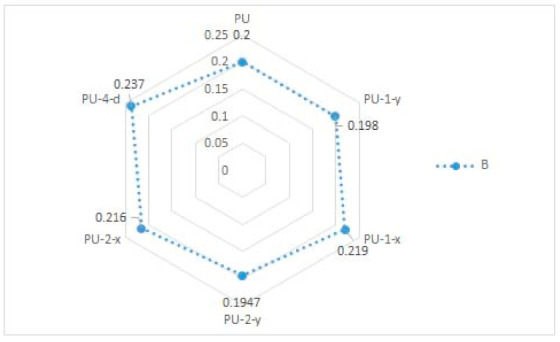
Bending stiffness (B) of polyurethane materials.

**Figure 10:**
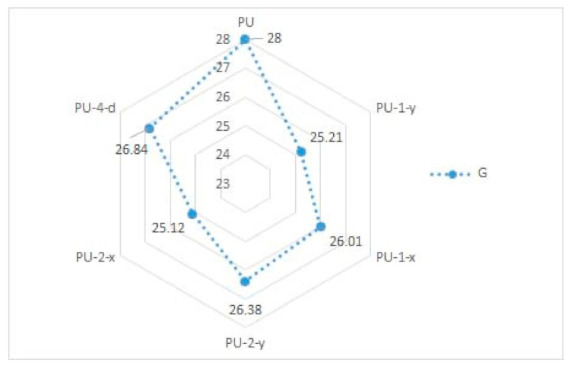
Shear stiffness (G) of polyurethane materials.

In Table 2 and Figure 5 and Figure 6, results of the measured mechanical properties (WC, RC, MIU, and SMD) of polyurethane materials without ultrasonic joints are given. In Figure 7, Figure 8, Figure 9 and Figure 10, a comparison of the measured mechanical properties (WT, EMT, B, and G) of polyurethane materials with and without ultrasonic joints is given.

As can be seen from Table 2, the compressional energy of polyurethane material was 0.046 N·m^−1^, with a coefficient of variation of 13.5%. Its compressional resilience was 81.02%, with a coefficient of variation of 11.13%. The coefficient of friction (MIU) and surface roughness (SMD) describe the material’s friction properties and surface contour. The measured value of the coefficient of friction was 1.064, while the value of surface roughness was 0.221 µm.

The tensile energy of the six polyurethane materials was in the range of 5.3–5.8 N·m^−1^ (Figure 7). As can be seen, the polyurethane material with two joints in the *y*-direction (i.e., sample designated as PU-2-y) had the highest value of tensile energy. The values of the tensile energy of the remaining materials were quite similar. The increase in tensile energy for materials with joints, in comparison to the polyurethane material without ultrasonic joints, was 2–8%. Figure 8 presents the results of the tested extension of polyurethane materials. As can be seen from the figure, the extension of materials was in the range of 2.2–2.45%. Among the tested polyurethane materials, the highest extension was again characteristic of the material with two joints in the *y*-direction (sample PU-2-y). Among the materials with one ultrasonic joint, the material with a joint in the *y*-direction exhibited higher extension (sample PU-1-y). This brought us to the conclusion that ultrasonic joints in the *y*-direction increased the overall extension of the designed polyurethane material. According to the obtained results, there were small differences between the values of bending stiffness of ultrasonically joint polyurethane materials (Figure 9). This can be explained by the change in thickness of polyurethane material in the area of the joint. Another reason for such behavior is a change in the structure of the material at the joint point, which prevented the material from slipping. The results of the shear stiffness (Figure 10) show that the polyurethane material with an ultrasonic joint had lower shear stiffness in comparison to the material without a joint. The reason was the rigidity of the ultrasonic joint, which prevented the shear of polyurethane material.

### 3.2. Measured Thermal Properties of PICs

**Figure 11:**
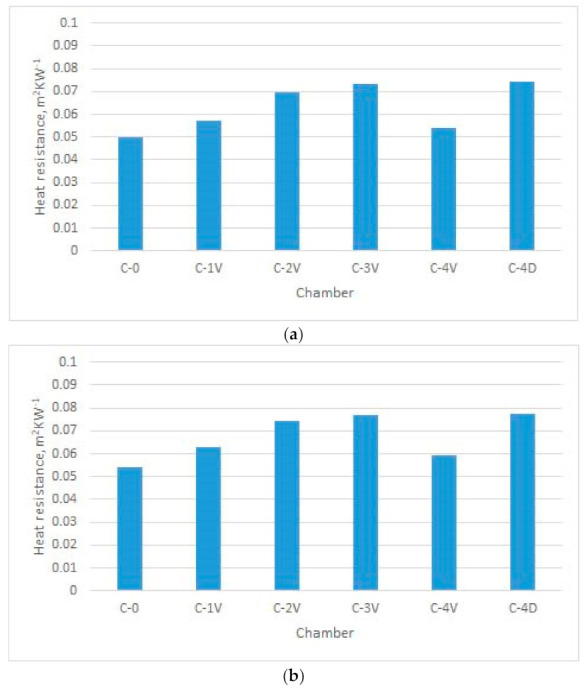

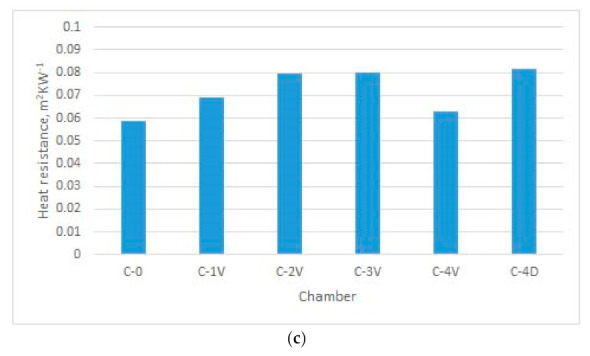
Heat resistance of designed noninflated PICs at different ambient temperatures (T): (**a**) T = 20 °C; (**b**) T = 15 °C; (**c**) T = 10 °C.

**Figure 12:**
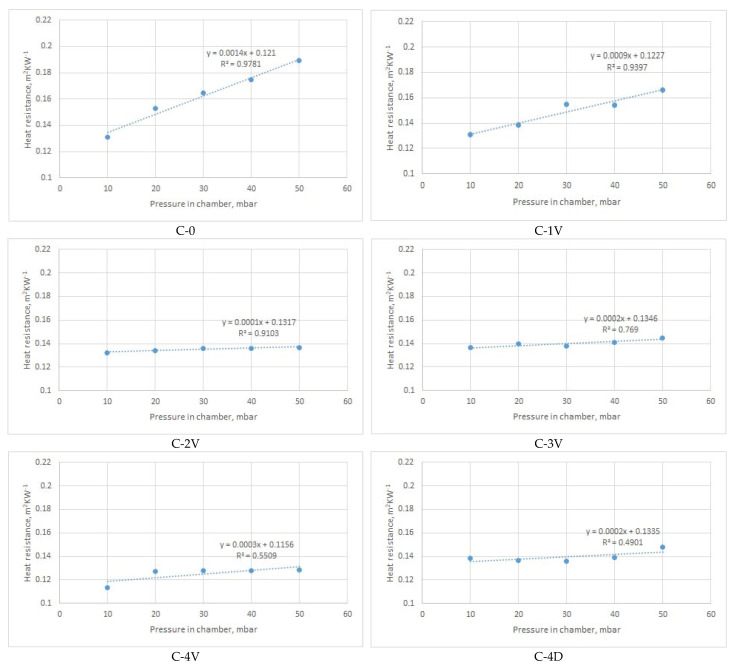
Dependence of heat resistance and air pressure of inflated PICs s at a constant ambient temperature of 20 °C.

**Figure 13:**
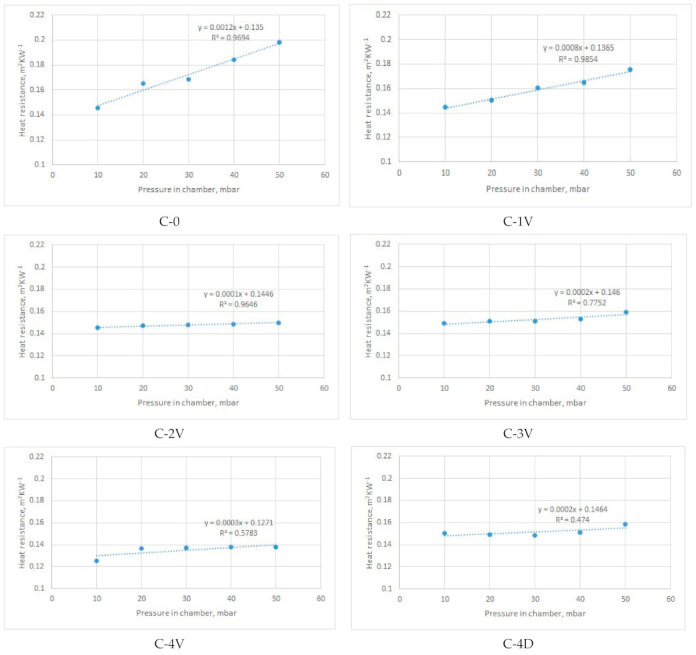
Dependence of heat resistance and air pressure of inflated PICs at a constant ambient temperature of 15 °C.

**Figure 14:**
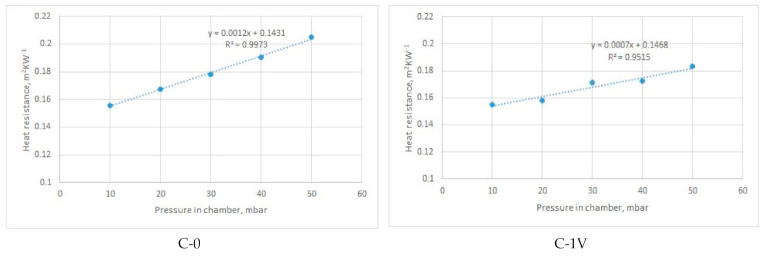

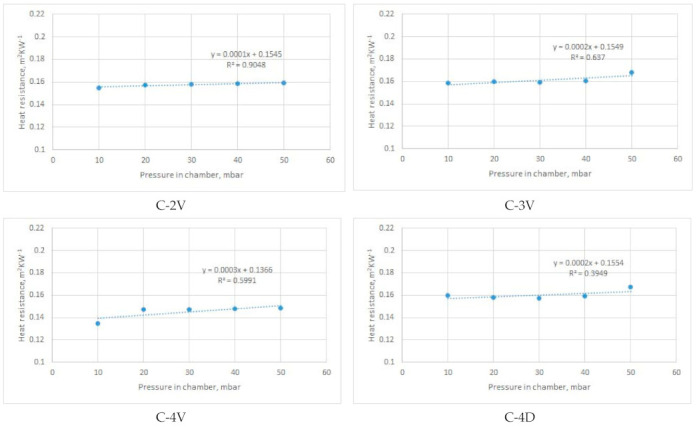
Dependence of heat resistance and air pressure of inflated PICs at a constant ambient temperature of 10 °C.

Figure 11, Figure 12, Figure 13 and Figure 14 show the results of the measured heat resistance of designed PICs at different pressures and temperatures.

In this study, a hot plate was used to test the heat resistance of six designed PICs. The PICs were tested under different pressures (0, 10, 20, 30, 40, and 50 mbar) and at different temperatures established in the climate chamber (20 °C, 15 °C, and 10 °C). Upon blowing air into the PIC, the pressure in the PIC increased. This means that there was more air in the PIC at a pressure of 50 mbar than at, for example, a pressure of 30 mbar. The amount of air in the PIC depended on its design. The measured heat resistance of designed PICs was from 0.0496 to 0.0815 m^2^·K·W^−1^ when air was not blown into the chambers (i.e., for nonactivated PICs), and from 0.1309 to 0.2047 m^2^·K·W^−1^ when air was blown into the chambers (i.e., for activated PICs).

The heat resistance of nonactivated PICs (i.e., at 0 bar of air pressure) was in the range of 0.049 to 0.082 m^2^·K·W^−1^. The influence of the design can be well seen in Figure 11. Specifically, under all three ambient temperatures (i.e., T = 10 °C, T = 15 °C, and T = 20 °C), the simplest designed PIC (i.e., the chamber without joints, designated as C-0), provided the lowest resistance to heat transfer. The *R_ct_* values were 0.049, 0.054, and 0.058 m^2^·K·W^−1^, respectively. This PIC was followed by the PIC with four vertical joints (designated as C-4V). The highest resistance to heat transfer was achieved by PICs with four diagonal joints and three vertical joints (chambers C-4D and C-3V).

It is interesting to note the changes in the heat resistance of PICs after air was blown into the chambers. Accordingly, the results shown in Figure 12 indicate that the heat resistance of the PIC without an ultrasonic joint (chamber C-0), which in the noninflated state had the lowest value, changed significantly. Already at a pressure of 20 mbar, the heat resistance of PIC C-0 became highest among the tested PICs (0.1531 m^2^·K·W^−1^). The reason for this significant change in the heat resistance of PIC C-0 was the different construction of the PIC. Specifically, the mentioned PIC was the only one among the six tested PICs that did not have an ultrasonic joint. In this PIC, even at a pressure of 20 mbar, a larger amount of air was blown due to the joints that, in other PICs, prevented the chamber from inflating. In this way, a smaller amount of air could be inflated into the chambers.

The PIC with four vertical joints (designated as C-4V) had the lowest heat resistance among all designed PICs. This refers to all investigated pressures and all three ambient temperatures. The reason for such performance can be found in the PIC design. More specifically, four vertical joints disabled the inflation of a higher amount of air for defined pressures.

Measurements of heat resistance were also performed at lower ambient temperatures, i.e., at 15 °C and 10 °C. Due to technical limitations, the temperature of 10 °C was the lowest temperature that could be established and kept constant during the entire measurement cycle in the existing climatic chamber. With these changes in ambient temperature, the heat resistance of all samples increased. Concerning the heat resistance of the PICs at a temperature of 20 °C, the heat resistance of PICs at 15 °C increased to 11%, and that at 10 °C increased to 19%.

Considering the results obtained, an intelligent garment with active thermal protection can benefit from the use of TEIC with four diagonal joints (C-4D). Satisfactory options are also the polyurethane chambers with two ultrasonic joints at a distance of 80 mm (C-2V) and with four ultrasonic joints at a distance of 60 mm (C-4V). All named PICs at a pressure of 50 mbar had a lower heat resistance than the other designed PICs. As a result, the mentioned TEIC would retain a smaller amount of heat next to the body in colder conditions, which would not fulfil its basic purpose. A PIC without an ultrasonic joint (designated as C-0) would not be suitable for practical application. Although it had an extremely high heat resistance when compared to the other tested PICs, it is very unsuitable for use because it inflated significantly, even at low pressures. This would negatively affect wear comfort and limit body movements. The remaining PICs had relatively similar values of heat resistance. Among them, the single-ultrasound PIC (C-1V) also inflated significantly and would affect both wear comfort and body movement.

Figure 12, Figure 13and Figure 14present the average values of measured heat resistance. The measuring device (sweating guarded hotplate) calculated the average value on the basis of 30 measurements obtained in short intervals. Since the device is highly precise and was located in the climatic chamber with stable conditions (relative humidity, air temperature, air velocity), the coefficients of variation of measured heat resistance were rather low. Specifically, the coefficients of variation (CV) of heat resistance of PICs measured in different environmental temperatures were as follows:

- for T = 20 °C: CV = 0.12–1.12%;
- for T = 15 °C: CV = 0.15–1.13%;
- for T = 10 °C: CV = 0.12–1.56%.

Figure 12, Figure 13and Figure 14 give the linear regression equation and coefficients of determination describing the proportion of variance in the heat resistance that is predic from the pressure in the chamber. As can be seen from the figures, high values of the coefficient were characteristic for PICs C-0, C-1V, and C-2V at all three ambient temperatures (20 °C, 15 °C, and 10 °C). According to the results, the remaining PICs exhibited moderate to weak correlation (the weakest one was for PIC C-4D at all temperatures).

In a previous study, the researchers showed that the ratio of thermal insulation of maximum activated TEIC was almost three times higher than that of nonactivated TEIC [17]. To compare results of the heat resistance of designed PICs, the ratios of maximum activated and nonactivated PICs were calculated and are presented in Table 3.

Table 3 shows that the observed ratio was the highest for the PIC without a joint (C-0). The range was from 3.47 (at 10 °C) to 3.82 (at 20 °C). This outcome was to be expected. The largest amount of air was blown into this PIC because it did not have a joint that would prevent the PIC form expanding. The ratio for the PIC with one joint (C-1V) was 2.65–2.90. Even with this PIC, a larger amount of air was blown. For other PICs, the ratio was about 2, indicating that the amount of blown air was approximately the same.

## 4. Conclusions

The experiment described in this paper was aimed at investigating the mechanical properties of polyurethane materials used to design polyurethane inflated TEIC, which serves as an adaptive thermal layer in intelligent clothing. Testing of their efficiency considering the thermal protection was also carried out. Throughout the experiment, the influence of the direction and number of ultrasonic joints on the mechanical properties of polyurethane material was determined, especially in terms of the increase in compressional resilience and tensile energy. From the presented results, it can be concluded that the design and construction of PICs are extremely important for the preservation of heat inside the garment. Their optimal choice can greatly affect the wear comfort, especially in a cold environment. The results indicate that a PIC with four diagonal ultrasonic joints is optimal for use within thermally adaptive clothing. In contrast, a PIC without joints is not recommended for practical application, as it would decrease the level of wear comfort and limit body movement. The results also show that the ratios of maximum activated and nonactivated PICs were in the range of 1.92 to 3.82.

It is expected that the outcomes of this study will be used when designing specific sport garments that will enhance the performance of top athletes in cold environments.

## Figures and Tables

**Table 1:** Design of polyurethane insulation chambers (PICs). ID, identifier.

No.	PIC ID	Dimension	Description
1	C-0	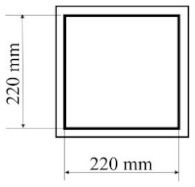	- joint type: ultrasonic - number of joints: 0 - position of joints: n/a - length of joint: n/a
2	C-1V	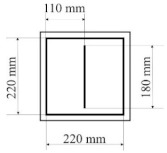	- joint type: ultrasonic - number of joints: 1 - position of joints: in the middle - length of joint: 180 mm
3	C-2V	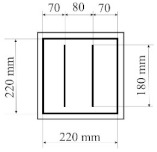	- joint type: ultrasonic - number of joints: 2 - position of joints: two joints with a spacing of 80 mm - length of joint: 180 mm
4	C-3V	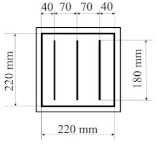	- joint type: ultrasonic - number of joints: 3 - position of joints: three joints with a spacing of 70 mm - length of joint: 180 mm
5	C-4V	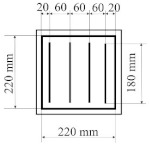	- joint type: ultrasonic - number of joints: 4 - position of joints: four joints with a spacing of 60 mm - length of joint: 180 mm
6	C-4D	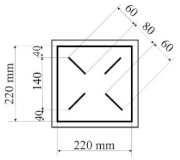	- joint type: ultrasonic - number of joints: 4 - position of joints: diagonal with break - length of joint: 60 mm

**Table 2:** Mechanical properties of polyurethane material without ultrasonic joint. WC, compressional energy; RC, compressional resilience; MIU, coefficient of friction; SMD, surface roughness.

Property	Index	Average Value	Coefficient of Variation, %
Compressional energy, N·m^−1^	WC	0.046	13.50
Compressional resilience, %	RC	81.02	11.13
Coefficient of friction, dimensionless	MIU	1.064	5.71
Surface roughness, μm	SMD	0.221	3.43

**Table 3:** The ratios of maximum activated and nonactivated PICs.

	Ambient Temperature		
PIC ID	20 °C	15 °C	10 °C
C-0	3.82	3.67	3.47
C-1V	2.90	2.78	2.65
C-2V	1.92	2.01	1.99
C-3V	1.97	2.00	2.09
C-4V	2.06	2.03	2.06
C-4D	1.99	2.04	2.05

## Data Availability

Data available in a publicly accessible repository.
